# Changing patterns and influencing factors of involuntary admissions following the implementation of China’s mental health law: A 4-year longitudinal investigation

**DOI:** 10.1038/s41598-019-51980-6

**Published:** 2019-10-24

**Authors:** Hua-Jian Ma, Bin Xie, Yang Shao, Jing-Jing Huang, Ze-Ping Xiao

**Affiliations:** Shanghai Mental Health Center, Shanghai Jiao Tong University School of Medicine, Shanghai, 200030 P.R. China

**Keywords:** Health policy, Palliative care, Epidemiology, Quality of life, Risk factors

## Abstract

Involuntary admission (IA) is limited to particular situations in mental health laws to protect patients from unnecessary coercion. China’s first national mental health law has been in effect since 2013; however, the status of IA has not been sufficiently explored. To explore the changing patterns of IA since the clinical application of the IA criteria specified in the new law, an investigation of IA status was undertaken in 14 periods (each period lasting for one month from 05/2013 to 05/2017) in the tertiary specialized psychiatric hospital in Shanghai. The socio-demographic and clinical characteristics of 3733 patients were collected. The differences among IA rates in different periods were compared, and the characteristics of patients who were and were not involuntarily admitted were analysed. Multiple logistic regression analysis was used to clarify the independent variables of IA. The IA rate dramatically decreased after the implementation of the new law, while the overall trend gradually increased. The implementation of the IA risk criteria is effective, but IA is still common in China. The medical factors influencing IA following the implementation of the new law are similar to those in previous studies at home and abroad. Non-medical factors might be the main causes of the high IA rates in Chinese psychiatric institutions.

## Introduction

Although it is a controversial topic because of the infringement on basic human rights and low levels of treatment satisfaction^1–3^, involuntary hospital admission is still a necessary measure in the management of mentally ill individuals who lack insight^4,5^. Mental health services in China were mostly provided by specialized psychiatric hospitals, including all kinds of psychiatric patients. Clinical practice in such institutions was based on diagnostic criteria such as the International Classification of Diseases (ICD-10) and therapeutic guidelines. The entire system was organized as more medically or clinically oriented than patients’ rights oriented. Previous studies reported that the IA rate in China was much higher than that in Western countries^6–9^. This difference was attributed to both social factors and, importantly, the lack of national legislation or standards regarding involuntary psychiatric hospitalization.

China’s first national mental health law – the Mental Health Law of the People’s Republic of China – was enacted on May 1st, 2013, after 27 years of debate and wide-ranging discussion at different levels^10^. Especially after the ratification of the International Covenant on Civil and Political Rights (ICCPR) and the International Covenant on Social, Economic and Cultural Rights (ICSECR) in the mid-1990s, coercion involving psychiatric services in China received more attention. Later, drafts of the law referred to international documents such as the United Nations General Assembly (46/119)^11^ and Mental Health Care Law: Ten Basic Principles^12^. Minimum restrictions, autonomy, and informed consent were largely reflected in this legislation.

**Figure 1:**
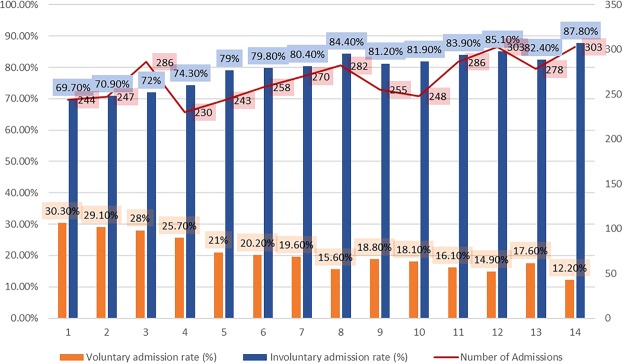
Histogram showing the pattern of IA, including the voluntary admission rate (%), involuntary admission rate (%) and number of admissions each month.

Given the complex factors involved, such as the need to protect the autonomy of mentally ill individuals, the traditions of China’s mental health services, and other socio-cultural variables, the drafters of the law replaced the broader ‘need for treatment’ criteria with much stricter risk-based criteria. The latter had been pilot-tested in local jurisdictions around China before being included in the national law. Specifically, ‘…the medical facility may impose inpatient treatment if the individual meets one of the following conditions: (1) self-harm in the immediate past or risk of self-harm; (2) behaviour that harmed others or endangered the safety of others in the immediate past or risk to the safety of others’^10^. In limiting involuntary admission to particular situations, the national law aimed to reduce the risk of infringing on the rights of patients and the occurrence of unreasonable IA in China.

Before the enactment of the new law, there were several investigations of the IA patterns in China, all of which found that IA is common in Chinese psychiatric hospitals. A multi-centre survey in 2002^6^ involving 17 cities and 2333 patients indicated that only 18.5% of psychiatric patients were voluntarily admitted. Another study of 161 patients in 2012 found an IA rate of 53.1%^7^. The IA rates in other investigations with small sample sizes were generally above 60%^13^. These studies also found that IA in China is associated with factors similar to those in Western countries^6,7,13,14^, including aggressive behaviour, poor social functioning, and lack of insight. However, the situation since the new law took effect is still unclear.

Shanghai, a city with relatively abundant medical resources, was the first area in China to enact local mental health regulations. A ‘need for treatment criterion’ for IA in this legislation had been applied for more than ten years before the implementation of the new, much more restrictive risk-based criteria. The change in IA legislation in Shanghai was significant and far-reaching. We can learn more about the IA pattern from an investigation in Shanghai because Shanghai is a typical city with a clear and dramatic shift in IA legislation. The present study analyses the rate of IA and identifies the factors associated with IA following the new law’s implementation in Shanghai. This study may provide supporting evidence to improve the design of the policy system and help explore possible trends and directions for future research in this area.

## Results

Of the 3864 patients admitted to the hospital during the study period, 3733 (96.6%) met the study criteria and participated in the study. Among the 3733 participants in the study, 2978 (79.8%) patients were involuntarily admitted, and 755 patients (20.2%) were voluntarily admitted. Male patients accounted for 44.7% (n = 1669) of all admissions and 44.3% (n = 1319) of involuntary admissions. No relationship was found between gender and admission status (p = 0.308). The mean age was 42.39 years (standard deviation [SD] 18.95). A diagnosis of schizophrenia was the most common (52.0%, N = 1940), followed by mood disorders (30.2%, N = 1127).

### Description of the voluntary and involuntary admission patterns

The pattern of voluntary and involuntary admission status is shown in Table 1 and Fig. 1. In general, the IA rate rose over time. It reached the highest point (87.8%) 49 months after the new law took effect, and the lowest rate of IA (69.7%) occurred in the first month after the law took effect.

**Table 1:** Pattern of IA frequency.

Serial number	Time period after the legislation	Voluntary admission (n = 755)		Involuntary admission (n = 2978)	
		N	%	N	%
1	1^st^ month (2013.05.01–2013.05.31)	74	30.3	170	69.7
2	2^nd^ month (2013.06.01–2013.06.30)	72	29.1	175	70.9
3	3^rd^ month (2013.07.01–2013.07.31)	80	28.0	206	72.0
4	4^th^ month (2013.08.01–2013.08.31)	59	25.7	171	74.3
5	5^th^ month (2013.09.01–2013.09.30)	51	21.0	192	79.0
6	6^th^ month (2013.10.01–2013.10.31)	52	20.2	206	79.8
7	7^th^ month (2013.11.01–2013.11.30)	53	19.6	217	80.4
8	8^th^ month (2013.12.01–2013.12.31)	44	15.6	238	84.4
9	9^th^ month (2014.01.01–2014.01.31)	48	18.8	207	81.2
10	10^th^ month (2014.02.01–2014.02.28)	45	18.1	203	81.9
11	11^th^ month (2014.03.01–2014.03.31)	46	16.1	240	83.9
12	12^th^ month (2014.04.01–2014.04.30)	45	14.9	258	85.1
13	30^th^ month (2015.10.01–2015.10.31)	49	17.6	229	82.4
14	49^th^ month (2017.05.01–2017.05.31)	37	12.2	266	87.8

### Demographic and clinical characteristics of the voluntary and involuntary admission groups

The demographic and clinical characteristics of the 755 voluntarily admitted patients were compared to those of the 2978 involuntarily admitted patients (Tables 2 and 3). The IA patients were significantly more likely to be under 44 years old, to be unemployed, to be single (unmarried, divorced or widowed), to have health insurance, to lack insight, to have been hospitalized more times and to have been diagnosed with schizophrenia.

**Table 2:** Demographic and clinical characteristics of the two groups (1).

	Voluntary admission (n = 755)		Involuntary admission (n = 2978)		Statistics		
	N	%	N	%	X^2^	df	*P*
**Gender: Male**	350	46.4	1319	44.3	1.040	1	0.308
**Age (years)**					22.057	2	0.000
≤44	479	63.4	1764	59.2			
45–59	167	22.1	559	18.8			
≥60	109	14.4	655	22.0			
**Occupation: unemployed**	507	67.2	2181	73.2	11.063	1	0.001
**Marital status**					31.053	2	0.000
Single	354	46.9	1422	47.8			
Married/remarried	357	47.3	1189	39.9			
Divorced/widowed	44	5.8	367	12.3			
**Family history**	207	27.4	810	27.2	0.014	1	0.904
**Health insurance**	334	44.2	1599	53.7	21.567	1	0.000
**Referring persons**					0.616	1	0.433
Relatives	748	99.1	2490	98.7			
Police and social affairs	7	0.9	38	1.3			
**Risk behaviours**							
Violent behaviours towards others	40	5.3	794	26.7	158.448	1	0.000
Suicidal or self-injurious behaviours	113	15.0	1041	35.0	112.687	1	0.000
Risk of self-harm	359	47.5	2490	83.6	433.432	1	0.000
Risk to the safety of others	96	12.7	1191	40.0	198.391	1	0.000
**Insight**							
None	238	31.5	2266	76.1	674.936	2	0.000
Partial	318	42.1	605	20.3			
Complete	199	26.4	107	3.6			
Initial diagnosis*					396.042	3	0.000
F0*	17	2.3	377	11.3			
F2*	225	29.8	1715	57.6			
F3*	361	47.8	766	25.7			
Other diagnosis	152	20.1	160	5.4			

^*^Initial diagnosis was made in the outpatient department as soon as the psychiatrists finished assessing the conditions of the patients. F0: Organic, including symptomatic, mental disorders; F2: Schizophrenia, schizotypal and delusional disorders; F3: Mood [affective] disorders.

**Table 3:** Demographic and clinical characteristics of the two groups (2).

	Voluntary admission (n = 755)		Involuntary admission (n = 2978)		Statistics	
	Mean	SD	Mean	SD	Z	P
Length of illness (months)	107.71	109.68	126.69	139.02	−1.210	0.226
Number of hospitalizations	1.03	2.19	2.14	3.89	−7.463	0.000

### Predictors of admission status

Table 4 presents the results of a forward stepwise multivariate logistic regression analysis that simultaneously considered all the factors significantly related to involuntary admission, which was the dependent variable (Tables 2 and 3). The independent variables entered in the model were age, occupation, marital status, insurance, risk behaviours, insight, initial diagnosis, and number of hospitalizations. Predictors of involuntary admission in this model were under age 44 or above age 60, single status, poor insight, diagnosis of schizophrenia or other psychotic illness, risk behaviours, and a higher number of hospitalizations. The R^2^ of this model was 0.489, which means that 48.9% of the variance in admission status could be explained by variables in this model.

**Table 4:** Independent contributors to IA status (multiple logistic regression analysis).

	P-value	Odds ratio	95% C.I.
**Total R** ^**2**^ ** = 0.489, p < 0.001**			
**Age (years)**			
≤44	0.026	1.000	—
45–59	0.825	1.034	0.771–1.385
≥60	0.011	1.619	1.119–2.344
**Marital status**			
Single	0.023	1.000	—
Married/remarried	0.840	0.974	0.756–1.255
Divorced/widowed	0.014	1.735	1.119–2.689
**Insight**			
None	0.000	1.000	—
Partial	0.000	0.335	0.265–0.423
Complete	0.000	0.147	0.104–0.207
**Number of hospitalizations**	0.000	1.111	1.060–1.165
**Risk behaviours: Violent behaviours towards others**	0.000	4.815	3.220–7.201
**Risk behaviours: Suicidal or self-injurious behaviours**	0.000	2.917	2.259–3.768
**Risk behaviours: Risk of self-harm**	0.000	9.597	7.533–12.226
**Risk behaviours: Risk to the safety of others**	0.000	3.254	2.412–4.391
Initial diagnosis*			
F2*	0.000	1.000	—
F0*	0.144	1.597	0.852–2.994
F3*	0.000	0.467	0.363–0.602
Other diagnosis	0.000	0.317	0.225–0.449
**Constant**	0.105	0.795	—

*Initial diagnosis was made in the outpatient department as soon as the psychiatrists finished assessing the conditions of the patients. F0: Organic, including symptomatic, mental disorders; F2: Schizophrenia, schizotypal and delusional disorders; F3: Mood [affective] disorders.

## Discussion

As the medical records system showed, the percentage of IA patients in Shanghai Mental Health Center (SMHC) was slightly higher than the average (81.5%)^6^ before the enactment of the national mental health law. The relatively high IA rate was related to the characteristics of SMHC, which is the only tertiary specialized psychiatric hospital in Shanghai and which provides first aid in psychiatric emergencies. Although the rate of IA declined sharply once the new legislation came into effect, the vast majority of psychiatric admissions remain involuntary. The IA rate after the new law is still much higher than those (3–50%) reported in Western countries and has risen gradually over time^3,8,15^.

In fact, the change in IA patterns after the new law came into effect in China was similar to that in Western countries. Research in Western countries found that legislation did not affect the IA rate as much as policy makers had hoped and that there might be a relatively weak effect of legislation on clinical practice or even a gradual return to pre-legislation status^15,16^; this may also be the case here. The IA rate declined rapidly under the influence of the new regulation; however, it rose gradually due to a lack of supporting measures following the law, such as detailed operational procedures on IA and sufficient community-based services. The mechanism had characteristics that were similar to those of the deinstitutionalization and reinstitutionalization of psychiatric services in some Western countries^17^.

Generally, the factors affecting the IA pattern could be divided into two categories: medical and non-medical. Medical factors refer to the conditions of psychiatric patients, such as their demographic and clinical features, while non-medical factors are the procedures and specific operations in the process of IA decision-making and even the traditional culture and development of the entire society.

As found in this study, the medical factors influencing IA were quite similar to those found in previous studies, such as poor insight^7,18^, history of hospitalization^19,20^, aggressive or self-harm behaviour^6,7,17,21^, and diagnoses of schizophrenia or other psychotic illness^4,9,15^. As stipulated in the dangerousness admission criteria of the new law, there was a significant association between patients’ aggressive or self-harm behaviour and IA. Except for the factors mentioned above, we found that single status was also significantly associated with patients’ IA status. Previous studies have shown that unmarried status is a risk factor for frequent hospitalization in patients with mental disorders^20,22,23^, as it may delay timely treatment due to insufficient daily care and increase the possibility of readmission, or it may indicate poorer social capability and more severe psychiatric symptoms of the patients, leading to necessary treatment. Additionally, we noted that patients under age 44 or above age 60 were more likely to be involuntarily admitted. This result may differ from the findings of prior studies, in which involuntarily admitted patients were comparatively older or younger in age^24,25^. This discrepancy may be partly attributed to the different diagnoses and treatment needs of the two age groups. The majority of younger patients are diagnosed with schizophrenia, which is correlated with IA^4,9,15^; however, elderly patients are mostly diagnosed with dementia, and the majority have poor insight, which is also significantly related to IA^7,18^. Furthermore, the ageing of the population may aggravate this situation.

Although 48.9% of the variance in admission status could be explained by medical factors in the final model (Table 4), more than half of the variance should be attributed to other factors. The medical factors in IA after the new law were generally similar to those found in studies at home and abroad. That is, non-medical factors were the main causes of the high numbers of IA patients in Chinese psychiatric institutions.

First, in terms of medical resources, the high IA rate may be partially explained by the relative lack of community-based mental health services and the stigma experienced by patients and their family members^26–31^. Second, on the legislative and operational levels, the phrase ‘risk to the safety of oneself/others’ in the law leaves considerable scope for discretion because there are no specific operational procedures or detailed regulations on the criteria, such as clarification of the timeframe considered (is it ‘imminent’ risk or prior risk?) or of the severity of the risk (is it mild, moderate or severe risk?)^32,33^, which may cause misuse or abuse of the criteria by psychiatrists^34,35^. Furthermore, research has identified different interpretations of IA criteria among psychiatrists even though there are clear regulations in the local legislation^34,36,37^, which means that psychiatrists may be more inclined to make decisions according to their professional judgement but not the legal provisions^34,36^. Third, psychiatrists play central roles in balancing the interests of the public with protecting the legal rights of mental disorder patients^38–42^, especially regarding circumstances in which patients do not present significant signs or clear risk of harming themselves or others^34,40^. Moreover, affected by traditional culture and legislation, Chinese families are burdened with the huge responsibility and obligation of taking care of and managing patients^13,43,44^. When a patient does not show a clear risk of harm to himself/herself or others, psychiatrists might practise ‘defensive psychiatry’ (i.e., ‘a practice of decision-making that limits the psychiatrist’s liability while protecting patients and others from potential harm’)^42,45^ in response to the stubbornness of relatives who may be more likely or reluctant to seek IA services.

In summary, detailed regulations on IA risk criteria and appropriate training of clinicians are necessary to control the effects of non-medical factors on the IA rate^20,46–48^. Although this is an epidemiological investigation and does not include an evaluation of any particular intervention, it does pave the way for future research and policy proposals on the IA process (i.e., the formulation of a detailed operational method and flow to improve the implementation of the IA criteria). To improve the implementation of the new criteria, we are developing a new IA assessment scale and will present it in our other articles.

## Methods

### Design and participants

This investigation was designed to identify patterns of IA following the implementation of the new risk criteria and to lay the groundwork for formulating an IA assessment process, which would be used to evaluate the appropriateness of IA for psychiatric patients. This study was conducted in the tertiary specialized psychiatric service system in Shanghai because such hospitals provide most of the IA services to residents. Emergencies and patients needing IA services are sent to tertiary specialized psychiatric hospitals first, while the secondary and primary psychiatric hospitals usually provide services to outpatients, the community and individuals with chronic mental disorders. One tertiary specialized psychiatric hospital and 19 secondary specialized psychiatric hospitals were operating in Shanghai. SMHC is the only tertiary specialized psychiatric hospital with 24-hour outpatient services in Shanghai, and it is also the largest psychiatric hospital in China, receiving more than 2000 outpatient visits per day and containing approximately 990 psychiatric inpatient beds.

A total of 3864 patients were consecutively admitted to SMHC in the first 12 months and in the 30th and 48th months following the implementation of the new mental health law. The inclusion criteria were as follows: the participants were adult inpatients (18 years old and above) in SMHC during the study periods; the patients were hospitalized for more than one week; and the patients (or their guardians if necessary) could understand the content of the interview and sign a written informed consent form. The exclusion criteria were as follows: individuals admitted compulsorily based on China’s revised Criminal Procedures Law (i.e., forensic patients); and patients’ medical records were incomplete or unavailable at the time of data collection. For patients admitted more than once over the follow-up periods, each admission was considered separately.

### Procedures and measures

Based on a review of the findings of related studies^15,16^, it was assumed that the IA rate would decline at the beginning of the implementation of the new legislation and remain relatively stable or rise gradually afterwards. Accordingly, this study was designed to investigate the IA pattern during the first year (i.e., from the 1^st^ to the 12^th^ months) and a few months in the next several years following the implementation of the new law (i.e., the 30^th^ and 48^th^ months depending on the situation of the study). The data were collected through the medical information system of the hospital so that the process of collecting a large amount of data would be more convenient and efficient. The inpatients’ socio-demographic characteristics, such as gender, age, occupation, marital status, and health insurance, were extracted and recorded, and the clinical information of the inpatients was collected simultaneously (i.e., family history, referring persons, risk behaviours, insight, initial diagnosis, length of illness, and number of hospitalizations).

Psychiatrists whose professional titles were attending psychiatrists or above (i.e., chief psychiatrists and associate chief psychiatrists) with more than 5 years of experience in clinical practice made the initial diagnoses, evaluated the insight of the patients and recorded their risk behaviours at the time of admission. Diagnoses were based on the International Classification of Diseases 10^th^ revision (ICD-10); if more than one diagnosis was listed, the principal (first) diagnosis was recorded. According to the prior records in the electronic medical information system of SMHC, inpatients who were diagnosed with organic mental disorders (F00-F09), schizophrenia-related disorders (F20-29), or mood disorders (F30-39) accounted for the majority of all inpatients. Therefore, ICD-10 diagnoses at admission were divided into 4 groups: F0, F2, F3, and others.

The psychiatrists were required to record the insight of patients at admission using three main levels (i.e., complete, partial and no insight) to obtain a preliminary understanding of the patients’ cognitive ability to evaluate their mental state. The patients’ insight was subjectively evaluated by the psychiatrists according to the following questions: *whether the patient believes that his/her abnormal experiences are symptoms; whether the patient believes his/her symptoms are attributed to a mental disorder; whether he/she believes that the disorder is psychiatric; whether he/she believes that psychiatric treatment might benefit him/her; and whether he would be willing to accept advice from a doctor regarding his/her treatment*. The risk behaviours of the patients were also categorized into 4 groups according to the criteria of the IA legislation: violent behaviours against others, suicidal or self-injurious behaviours, risk of self-harm, and risk to the safety of others. The psychiatrists would select the relevant items in accordance with the patients’ specific situations.

### Statistical analysis

The data were analysed using SPSS version 17.0 (SPSS Inc., Chicago, IL, USA). A descriptive analysis of the frequency of IA was conducted. The comparison of the basic demographic and clinical characteristics between voluntary and involuntary patients was performed with one-way ANOVA, the Mann-Whitney U test, and the chi-square test, as appropriate. Multiple logistic regression including all the independent variables that differed significantly between the two groups (voluntary and involuntary admission groups) was used to assess the independent contributors to involuntary admission. The level of significance was set at 0.05 (two-tailed).

### Ethical approval and informed consent

The Research Ethical Committee at the Shanghai Mental Health Center (IORG Number: IORG0002202; FWA Number: FWA00003065) approved the study. The director of the participating psychiatric hospital approved the collection of data from electronic information systems and case files at the institution. All the personal information of the patients and psychiatrists was properly protected in the analysis. All the research was performed in accordance with relevant regulations, and written informed consent was obtained from all the participants.

## Data Availability

The datasets generated during and/or analysed during the current study are available from the corresponding author on reasonable request.
